# Levitronix bilateral ventricular assist device, a bridge to recovery in a patient with acute fulminant myocarditis and concomitant cerebellar infarction

**DOI:** 10.5830/CVJA-2018-009

**Published:** 2018

**Authors:** Huang Yi-Fan, Hsu Po-Shun, Tsai Chien-Sung, Tsai Yi-Ting, Lin Chih-Yuan, Ke Hong-Yan, Lin Yi-Chang, Yang Hsiang-Yu, Huang Yi-Fan, Tsai Chien-Sung

**Affiliations:** Division of Cardiovascular Surgery, Department of Surgery, Tri-Service General Hospital, National Defense Medical Centre, Taipei, Taiwan, Republic of China; Division of Cardiovascular Surgery, Department of Surgery, Tri-Service General Hospital, National Defense Medical Centre, Taipei, Taiwan, Republic of China; Division of Cardiovascular Surgery, Department of Surgery, Tri-Service General Hospital, National Defense Medical Centre, Taipei, Taiwan, Republic of China; Division of Cardiovascular Surgery, Department of Surgery, Tri-Service General Hospital, National Defense Medical Centre, Taipei, Taiwan, Republic of China; Division of Cardiovascular Surgery, Department of Surgery, Tri-Service General Hospital, National Defense Medical Centre, Taipei, Taiwan, Republic of China; Division of Cardiovascular Surgery, Department of Surgery, Tri-Service General Hospital, National Defense Medical Centre, Taipei, Taiwan, Republic of China; Division of Cardiovascular Surgery, Department of Surgery, Tri-Service General Hospital, National Defense Medical Centre, Taipei, Taiwan, Republic of China; Division of Cardiovascular Surgery, Department of Surgery, Tri-Service General Hospital, National Defense Medical Centre, Taipei, Taiwan, Republic of China; Division of Cardiovascular Surgery, Department of Surgery, Taoyuan Armed Forces General Hospital, Taipei, Taiwan, Republic of China; Division of Cardiovascular Surgery, Department of Surgery, Tri-Service General Hospital Songshan Branch, Taipei, Taiwan, Republic of China

**Keywords:** entricular assist device, acute myocarditis, cerebellar infarction

## Abstract

We report on the case of a 27-year-old male who presented to our emergency room with chest tightness, dyspnoea and cold sweats. The 12-lead electrocardiogram showed diffuse ventricular tachycardia with wide QRS complexes. Troponin-I level was elevated to 100 ng/ml. The coronary angiogram showed good patency of all three coronary vessels, and acute fulminant myocarditis was suspected. The patient underwent cardiopulmonary resuscitation in the catheter room and high-dose inotropic support was initiated to stabilise his haemodynamic status. After resuscitation, the patient was in a coma and acute stroke was highly suspected. In addition, deteriorating cardiogenic shock with acute renal failure and pulmonary oedema were also detected. Due to haemodynamic compromise despite high-dose inotropic support, a Levitronix® bilateral ventricular assist device (Bi-VAD) was implanted on an emergency basis for circulatory support. Postoperative brain computed tomography revealed acute left cerebellar infarction. Because the patient had left cerebellar infarction with right hemiplegia, heart transplantation was contraindicated. Eventually, cardiac systolic function recovered well and the patient underwent successful Bi-VAD removal after a total of 18 days on Levitronix® haemodynamic support. He was weaned from the ventilator two weeks later and was discharged 10 days later.

In the past two decades, intra-aortic balloon pump and extra-corporeal membrane oxygenation (ECMO) have been predominantly used at our centre as a bridge, either to cardiac transplantation or to recovery in patients with decompensated heart failure.^1^,^2^ However, most patients die because of either ECMO-related morbidity or systemic malperfusion if cardiac function does not recover in time and cardiac transplantation is contraindicated in this period.^2^ In such patients, LevitronixR bilateral ventricular assist device (Bi-VAD) could provide temporary cardiac support for a much longer period than ECMO.^3^ Our experience with this case indicates that timely implantation of Bi-VAD can function as a bridge to recovery in patients with acute fulminant myocarditis, particularly when heart transplantation is contraindicated.

A 27-year-old man was brought to our emergency room with a history of chest tightness, dyspnoea and cold sweats that had manifested a few hours earlier. However, the symptoms did not ameliorate with rest. He denied any systemic disease, except a common cold one week earlier.

The 12-lead electrocardiogram (ECG) showed diffuse ventricular tachycardia with wide QRS complexes. Troponin-I levels were elevated to 100 ng/ml. An emergency coronary angiogram showed good patency of all three coronary vessels, and acute fulminant myocarditis was suspected.

His haemodynamic status suddenly deteriorated because of ventricular fibrillation shortly after the angiogram, and cardiopulmonary resuscitation was performed for 30 minutes. His vital signs were restored after initiation of high-dose inotropic support with multiple inotropic agents (dopamine: 15 mcg/kg/min, dobutamine: 15 mcg/kg/min, norepinephrine: 32 mcg/min and epinephrine: 1 mcg/min). Because his vital signs were unstable during the coronary angiogram, we did not perform a routine myocardial biopsy, which was necessary for the pathological diagnosis of myocarditis.

We had no choice but to send the patient back to the intensive care unit for stabilisation of the haemodynamic status. However, his level of consciousness did not improve (Glasgow coma scale score 3). Emergency brain computed tomography (CT) showed no intracranial haemorrhage, and magnetic resonance imaging (MRI) was contraindicated because infusion pumps were implanted. We were therefore unable to rule out acute stroke.

Meanwhile, 24-hour hypothermia therapy (HT) at approximately 34°C was employed for neurological protection in post-resuscitation circulatory shock. Transthoracic echocardiogram showed general hypokinesia of both ventricles (left ventricular ejection fraction 15–20%). Despite these interventions, acute pulmonary oedema and deteriorating liver and renal function with progressive oliguria ensued.

To avoid multiple organ dysfunction syndrome, a continuousflow LevitronixR CentriMag Bi-VAD (LevitronixR, Waltham, MA) was implanted via a median sternotomy under the guidance of transoesophageal echocardiography. First, the left heart vent tube was inserted from the right superior pulmonary vein into the left ventricular apex, and the arterial cannula was inserted into the ascending aorta. Second, the right heart vent tube was inserted into the right atrium, and the arterial cannula was inserted into the pulmonary artery (Fig. 1). Then pursestring sutures with non-absorbable retention sutures secured with tourniquets and spigots were tied around all the cannulae. The vital signs immediately stabilised with Bi-VAD support and the high-dose inotropic support was tapered off the next day (dopamine: 5 mcg/kg/min, dobutamine: 5 mcg/kg/min and norepinephrine: 3.7 mcg/min).

**Fig. 1:**
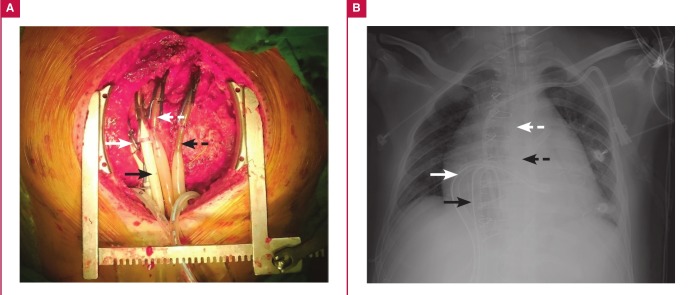
(A) Photograph of the cardiac operation and (B) a chest X-ray showing the left heart venous drainage tube (white arrow), the arterial perfusion tube (white dotted arrow), the right heart venous drainage tube (black arrow), and the arterial perfusion tube (black dotted arrow).

Because a Bi-VAD was inserted, systemic heparinisation therapy was administered through a peripheral line to maintain an activated clotting time (ACT) at 160–180 s using the Hemochron® Response ACT point-of-care testing system. Unfortunately, CT the following day showed an acute left cerebellar infarction (Fig. 2), which resulted in right hemiplegia. We assumed that the cerebellar infarction was caused by the cardiopulmonary resuscitation rather than VAD-related thrombus formation. To prevent post-infarct haemorrhage, we tapered the ACT to 140–160 s.

**Fig. 2:**
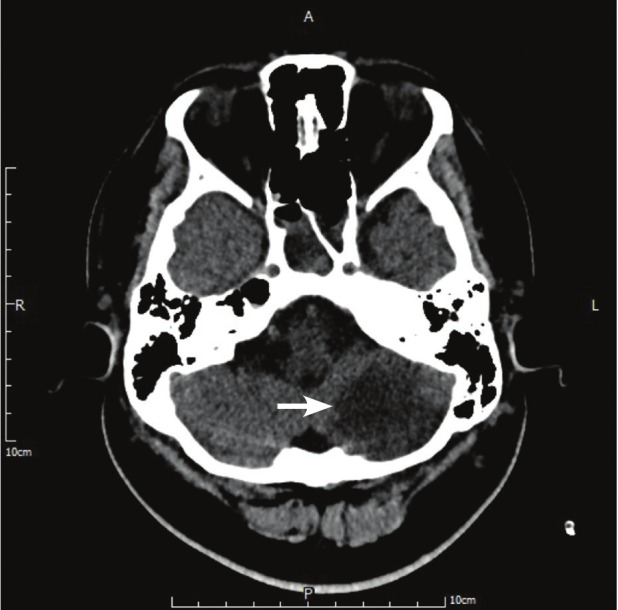
Hypodense defect indicates acute left cerebellar infarction (white arrow).

Three days later, the patient fully recovered from the coma, and his muscle power also improved. Daily echocardiography examinations showed progressive improvement of the cardiac systolic function.

When the left ventricular ejection fraction was approximately 50%, the Bi-VAD was weaned (right-VAD: 0.5 l/min; left-VAD: 0.8 l/min). The patient underwent successful Bi-VAD removal, after a total of 18 days on LevitronixR haemodynamic support. One week later, a tracheostomy was performed, and the patient was weaned from the ventilator a week later. The follow-up CT showed chronic encephalomalacia of the left cerebellum (Fig. 3).

The patient was discharged home 10 days later, after a total hospital stay of 42 days. During out-patient follow up, no signs of heart failure or device-related complications were noted.

**Fig. 3:**
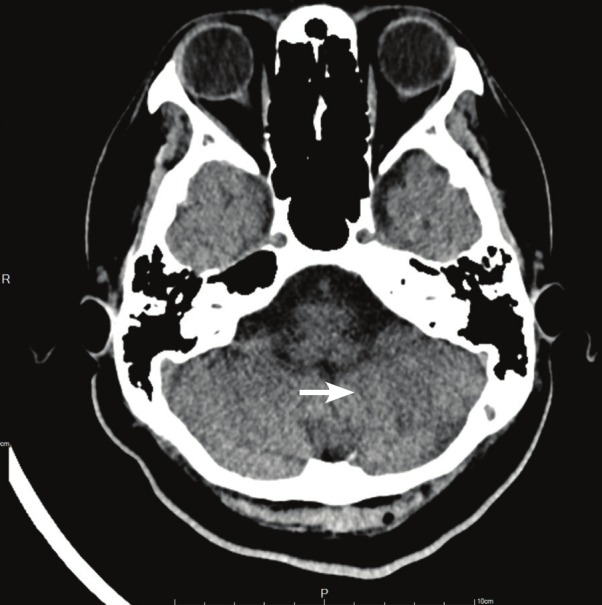
The initial hypodense lesion progressed to chronic encephalomalacia of the left cerebellum (white arrow).

## Discussion

Before the use of VADs became popular, ECMO played an important role in the haemodynamic support for decompensation in cases of acute heart failure at our centre.^1^,^2^ VADs provide substantially longer durability and considerably fewer complications than ECMO.^3^-^5^ According to the 2016 guidelines recommended by the International Society for Heart and Lung Transplantation, clinically severe symptomatic cerebrovascular disease may be considered a contraindication to transplantation.^6^ Our policy was to use VADs as a bridge, either to recovery or transplantation if the patient was able to recover from left cerebellar infarction with right hemiplegia.

After starting VAD, high-dose inotropic support could be tapered immediately (Table 1) to prevent vasoconstriction in the vital visceral organs. During the period with VAD support, both troponin-I levels and liver function progressively returned to normal ranges. In time, cardiac function improved and the VAD was successfully removed. Although renal function did not recover promptly and haemodialysis was necessary during the VAD period, the renal function did recover completely, albeit two weeks later after VAD removal. This means that timely VAD support could provide immediate circulatory support, enabling cessation of high-dose inotrope administration, which would cause vasoconstriction in the visceral organs, sequential ischaemia of the visceral organs, and consequent multiple organ failure.

**Table 1:** Clinical time course

	*Before VAD*	*POD1*	*POD2*	*POD3*	*POD6*	*POD9*	*POD12*	*POD15*	*POD18* *VAD removal*
Cardiac enzyme									
BNP (pg/ml)	350	518	553	2219	1974	584	590	405	363
CK (U/l)	1743	2609	3098	3245	800	219	63	53	45
CKMB (U/l)	136.6	67.8	–	–	–	–	–	–	–
Troponin-I (ng/ml)	90.06	94.02	60.70	33.65	6.31	0.68	0.22	0.11	0.05
Renal function									
BUN (mg/dl)	33	33	41	49	108	52	70	114	121
Creatinine (mg/dl)	3.4	3.6	4.6	4.7	7.7	5.3	3.8	3.6	3.1
Daily urine amount (ml)	0	128	250	545	1540	1120	1160	2550	2240
Liver function									
GOT (U/l)	214	722	1191	795	133	94	47	52	40
GPT (U/l)	81	416	633	706	372	200	66	60	64
Total bilirubin (mg/dl)	4.0	2.7	3.6	3.2	2.5	1.4	1.8	2.5	2.0
Inotropic agent									
Dopamine (mcg/kg/min)	15.0	5.1	5.1	3.0	1.8	2.9	4.7	4.4	7.6
Dobutamine (mcg/kg/min)	15.0	5.1	5.1	3.0	1.8	2.9	2.9	3.1	7.6
Epinephrine (mcg/min)	1.0	–	–	–	–	–	–	–	–
Norepinephrine (mcg/min)	32	3.7	–	–	2.0	1.6	1.2	–	–
VAD flow									
Right (l/min)		2.7	3.5	3.2	3.0	3.0	2.8	1.6	
Left (l/min)		4.5	5.0	5.0	5.0	5.0	4.8	2.6	
VAD revolutions per minute									
Right (/min)		3000	3000	2900	2900	2900	2700	1700	
Left (/min)		4100	4000	3700	3700	3700	3500	2000	

POD = post-operative day; VAD = left ventricular assist device; BUN = blood urea nitrogen; CK = creatinine kinase; CKMB = creatinine kinase MB coenzyme; GOT = glutamic-oxaloacetic transaminase; GPT = glutamic-pyruvic transaminase.

Currently, VADs can be categorised into two major types: pulsatile-flow and continuous-flow VADs. We chose the LevitronixR VAD for the following reasons. First, recent studies showed better outcomes with continuous-flow VADs than with pulsatile-flow ones. In addition, complications associated with continuous-flow VADs, especially bleeding and thromboembolism, are lower.^7^,^8^

Second, we expected recovery of the stunned myocardium after the acute myocardial infarction. Therefore, we abandoned use of the most advanced VADs, such as the HeartMate II9 or HeartWare,^10^ in which cannulation of the ventricular apex for drainage is necessary. We rather used the LevitronixR because we could cannulate the vent tube on the right superior pulmonary vein (Fig. 1) instead of the ventricular apex. The apical location would make surgical repair much more difficult, assuming that the viable but stunned myocardium could recover and the VAD could be withdrawn. We used a Snell-tie retention suture to securely fix both the vent and perfusion tubes. This also enabled a simple and quick closure of the cannulation wounds, which would facilitate the withdrawal procedure.

Third, although various kinds of VADs are available in Taiwan, not all are reimbursed by the National Health Insurance. In Taiwan, the LevitronixR costs approximately US$12 000, while the HeartMate II or HeartWare costs over US$170 000. Only the LevitronixR is covered by the National Health Insurance. Therefore, we opted to use LevitronixR instead of the HeartMate II or HeartWare because of economic considerations.

However, complications such as coagulopathy, thromboembolisation and mechanical failure are common with VAD use. Thromboembolisation after VAD implantation resulting in cerebrovascular events is devastating.^11^ There is no optimal treatment for stroke in patients implanted with a VAD. Supportive anticoagulation therapy rather than thrombolytic therapy, which is potentially associated with greater risk of haemorrhagic events after major surgery, is obviously essential treatment in this population.^12^

In our current protocol for heparinisation therapy, we prefer to maintain the ACT at approximately 160 s to avoid major, spontaneous bleeding disasters. If there are complications such as surgical bleeding or symptoms of coagulopathy (e.g. bloody sputum, massive gastrointestinal bleeding and large subcutaneous ecchymoses), we taper the heparin dose and maintain the ACT at approximately 140 s. If VAD-related thrombosis is suspected, we aim to prolong the ACT at 180–250 s.

In the present case, the pre-VAD CT scan showed no cerebral ischaemia, but the post-VAD CT scan showed acute left cerebellar infarction (Fig. 2). Judging by the chronology of events, the stroke event was suspected to be due to cardiopulmonary resuscitation rather than VAD-related embolisation. This is because a stroke event cannot usually be detected in the acute stage (especially within 24 hours) on CT. The only imaging examination to confirm acute-stage stroke is MRI, which was not practicable for this patient. Therefore we maintained the ACT at 140–160 s rather than > 180 s. Of course, post-infarct haemorrhage is another big concern. Eventually, we did not maintain the ACT at < 140 s because the CT scan showed no post-infarct haemorrhage.

In this case, the patient suffered from concomitant fulminant myocarditis and acute cerebellar infarction. It is useful to stabilise the infarct without ischaemic expansion or haemorrhagic transformation by maintaining the ACT at 140–160 s throughout the course. Our experience in this case indicates that short-term VAD use would be the first choice for mechanical circulatory support, not only because of much shorter surgery time but also cost-effectiveness, especially in patients with unconfirmed brain damage.

## Conclusion

LevitronixR VAD provides excellent short-term cardiac mechanical support for patients with severe symptomatic cerebrovascular disease. It offers patients an option, a bridge to recovery, that is not only cost-effective but also decreases cardiac trauma during potential removal.
